# Epigenetics in the pathogenesis of diabetic nephropathy

**DOI:** 10.3724/abbs.2021016

**Published:** 2021-12-23

**Authors:** Xue Li, Lihong Lu, Wenting Hou, Ting Huang, Xiangyuan Chen, Jie Qi, Yanjun Zhao, Minmin Zhu

**Affiliations:** 1.Department of Anesthesiology Fudan University Shanghai Cancer Center; Department of Oncology Shanghai Medical College Fudan University Shanghai 200032 China; 2.Department of Anesthesiology Shanghai General Hospital Shanghai Jiao Tong University School of Medicine Shanghai 200080 China; 3.Department of Anesthesiology Eye & ENT Hospital Fudan University Shanghai 200031 China

**Keywords:** diabetes, diabetic nephropathy, epigenetics, metabolic memory

## Abstract

Diabetic nephropathy (DN), which is a common microvascular complication with a high incidence in diabetic patients, greatly increases the mortality of patients. With further study on DN, it is found that epigenetics plays a crucial role in the pathophysiological process of DN. Epigenetics has an important impact on the development of DN through a variety of mechanisms, and promotes the generation and maintenance of metabolic memory, thus ultimately leading to a poor prognosis. In this review we discuss the methylation of DNA, modification of histone, and regulation of non-coding RNA involved in the progress of cell dysfunction, inflammation and fibrosis in the kidney, which ultimately lead to the deterioration of DN.

## Introduction

Diabetes, as a major medical problem in this century, threatens human health. About 92.4 million adults in China suffer from diabetes (accounting for 9.7% of the total adult population), and 60.7% of them have not been diagnosed or received relevant treatments [1]. More seriously, the incidence rate of diabetes has markedly increased in the last few decades around the world. According to statistics from the International Diabetes Federation, there were 463 million diabetic patients in 2019. It is estimated that the number of people with diabetes will increase to 700 million by 2045 [2].

Previously, in the Diabetes Complications and Control Trial (DCCT), patients with type I diabetes regulated their blood glucose level through receiving standard or intensive treatment. It has been demonstrated that the progression of microvascular complications is significantly reduced in patients with intensive treatment [3]. As a consecutive experiment of DCCT, the Diabetes Intervention and Complications Epidemiology (EDIC) trial showed that compared with patients who had received intensive treatment throughout the trial, the incidence of diabetes complications of the patients who had received standard treatment and switched to intensive treatment a few years later is still higher [ 4, 5]. Therefore, for some diabetic patients, especially those who start to strengthen blood sugar control after 8-11 years of illness, only applying measures to control blood sugar within the normal range cannot effectively prevent the occurrence of related cardiovascular complications [ 6– 9].This kind of hyperglycemic stress state that the body continues to maintain even if blood sugar returns to normal is defined as ‘hyperglycemia memory’ [ 10, 11].

**Figure 1:**
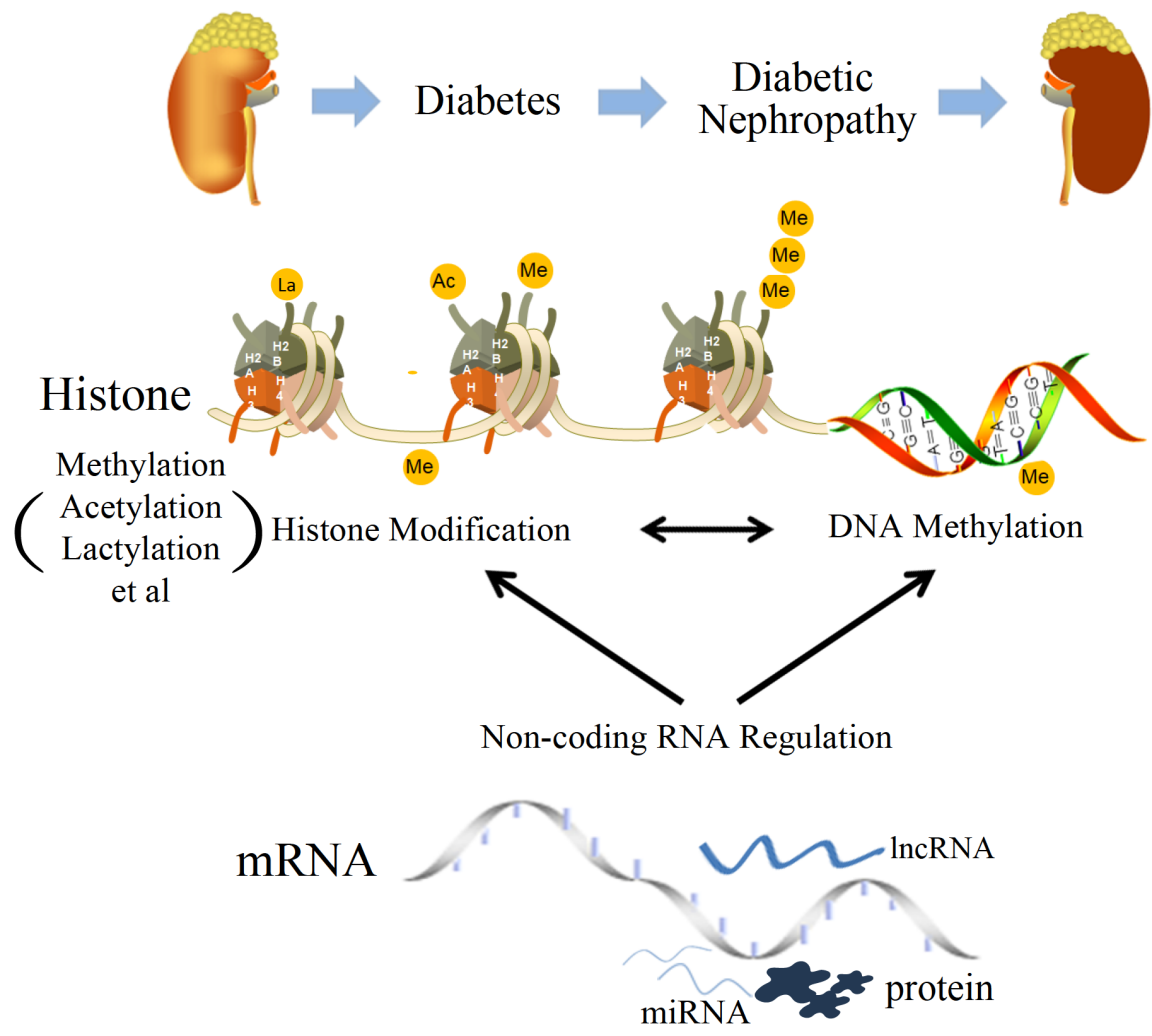
Figure1 The potential mechanisms of epigenetic regulation of diabetic nephropathy

Current studies showed that some signal transduction mechanisms in the diabetic state, including oxidative stress, advanced glycation end products (AGEs) receptor (RAGE) activation, tyrosine kinases, mitogen-activated protein kinases (MAPKs), protein kinase C (PKC), and nuclear factor kappa beta (NF-κB), participate in the development of high-glycemic memory [ 12– 17]. In addition, the AGEs and transforming growth factor-beta (TGF-β) produced in high-glycemic memory can have serious adverse effects on the target cells of renal damage [ 18– 21]. Whole-genome analysis denoted that genetic testing technology alone cannot make an accurate assessment of the risk of diabetes and its complications [22]. The discovery of ‘metabolic memory’ has become sound evidence of the mechanism of epigenetics with prolonged effects [23], which has attracted massive attention during the study of the pathogenesis of DN. Moreover, previous studies did not reveal a causal link between epigenetics and changes in gene sequences but did prove that epigenetics affects phenotypes [24], causing changes in external characteristics, revealing the interaction between genetic material and the environment, including DNA methylation, post-translational histone modifications, and non-coding RNA regulation ( Figure 1) [ 25– 29]. Epigenetic modification regulates relative gene expressions on one side, which enable the human body to respond quickly to the change in the surrounding circumstances, and guarantees to remember these reactions to establish a internal metabolic memory on the other side [30]. DN is a ubiquitous microvascular complication among diabetic patients, and the main pathogenesis of the end-stage renal disease. Its pathological characteristics include the thickened glomerular basement membrane (GBM), increased mesangial matrix, tubulointerstitial fibrosis, and podocyte loss [31]. The formation mechanism of DN is quite complicated, including genetic and non-genetic factors. High-throughput sequencing technology revealed that epigenetics regulates gene expression through a single or synergistic effect, which ultimately influences the occurrence and progression of DN [32]. Hence , epigenetics has been extensively studied in the microvascular endothelial damage caused by high glucose. So, studies on the mechanism by which epigenetic modifications mediate the morbidity and mortality of patients with diabetes and on its microvascular complications are of great practical significance [27].

Therefore, this article reviews the latest progresses in the research about epigenetics in the pathogenesis of DN.

### DNA Methylation

DNA methylation is catalyzed by the DNA methyltransferases (DNMTs), with the methyl group being transferred to the 5’ end of the cytosine residue in the dinucleotide cytosine guanine (CpG) [33]. Among these DNMTs, DNMT1 plays a role during the replication of DNA, while DNMT3a and DNMT3b mainly have the function of re-methylation during cell development [34]. DNA methylation directly interferes with the binding of the transcription complex in the promoter region or indirectly recognizes 5-methylcytosine, through the methyl-binding protein, to recruit the co-repressor to bind to the promoter region [35]. Hence, transcription disorders may cause abnormal methylation of key genes and consequently lead to some diseases [36]. During the development of DN from hyperglycemia, there are 694 hypomethylated CpG sites and 174 hypermethylated sites in the genome, including the inflammation of glucose metabolism, oxidative stress, mitochondrial stress and fat metabolism, which are involved in DN pathogenic gene locus [37]. Comparison of the type 1 diabetes patients with or without complications showed that DNA methylation of key genes changes over time [38], which provides direct evidence for a relationship between DNA methylation and hyperglycemia-induced metabolic memory.

Chronic oxidative stress is another crucial cause of DN [39]. Various studies have revealed that oxidative stress regulates DNA methylation and results in disease accordingly [ 40– 42]. Furthermore, the existence of metabolic memory exacerbates the production of reactive oxygen species (ROS) [43]. ROS, via modulating the activity of DNMT and damaging DNA, regulates methylation of key genes sequentially. Namely, ROS, as extremely reactive compounds, can produce many damaged sites in DNA. Superoxide dismutase 2 (SOD2), which is a key factor in anti-oxidative stress, can be inhibited by DNA methylation, thus mediating smooth muscle cell proliferation [44]. On the other hand, the abnormal methylation of SOD2 activates hypoxia inducible factor-1α (HIF-1α) [45], which inhibits HO-1-mediated control of mitochondrial dynamics, and prevents the overproduction of ROS [ 46, 47]. So, HIF-1α can exert a protective effect in DN.

The expression of DNMT1 is increased in mouse podocytes stimulated by high glucose [48], while this tendency can be attenuated after treatment with 5-azacytidine, an inhibitor of DNMT1, accompanied by a reduction of proteinuria and glomerular hypertrophy and an improvement of podocyte motility. Hence, DNMT1 may be a therapeutic target for protecting against DN podocyte injury [48]. In the kidneys of diabetic mice, the proximal tubular cells showed DNA hypomethylation of myo-inositol oxygenase (MIOX), which could be firmly bound by the transcription factor Sp1 on the gene promoter, thus mediating kidney damage progress [49]. Similar effects of abnormal DNA methylation models come under observation in podocytes cultured in a hyperglycemic environment [50]. The promoter region of matrix metalloproteinase (MMP)-9 also contains demethylated CpG sites, which can be induced by hyperglycemia, leading to the epithelial-mesenchymal transition (EMT) of podocytes [51]. The analysis of clinical samples of DN patients revealed that the demethylated promoter region of MMP-9 is not only correlated with the diagnostic indicators of DN positively, but also correlated with the glomerular filtration rate (GFR) negatively. This phenomenon denotes that the demethylation level of the promoter region of MMP-9 has a strong causal relationship with the pathophysiology of DN [52]. In addition, further research revealed that the connection between DNA methylation and histone modification could be mediated partially through methylcytosine-binding proteins, such as MECP2 and MBD2, which can recruit histone deacetylases to the methylated region [ 53, 54]. As a mark of repressive chromatin, DNA methylation could also dimethylate H3K9 through the interaction of G9a and DNMT1 with the replication complex [ 54, 55].

*In* *vivo* experiments have confirmed that change of the methylation level of differentially methylated regions of necrosis factor-alpha (TNF-α) with the use of dCas9-Tet1 system could alter TNF-α expression at the transcriptional level [56]. Moreover, TNF-α, produced by macrophages/monocytes [57], was proved to be related to the development of DN consequentially. In addition, it was reported that DNMT1 is up-regulated in peripheral immune cells of diabetic patients, which mediates the hypermethylation of the negative regulators of mTOR, causing the activation of the mTOR signaling pathway and resulting in the renal inflammation [58]. What’s more, in the leukocyte DNA methylation patterns of T2DM patients, the methylation of 77 CpG sites was found to be associated with a decrease in GFR [59]. Among these targets, Cdc42 GTPase activating protein (CdGAP), FK506-binding protein like (FKBPL), and activating transcription factor 6 beta (ATF6B) show consistent associations with the directionality of matching kidney tissue fibrosis. Moreover, CdGAP, FKBPL, and ATF6B have been reported to participate in mediating immune cell migration, inflammation and glomerulosclerosis [ 60– 62], indicating that the argument of DNA methylation in peripheral immune cells is a potential biomarker of DN progression.

### Histone Modification

Histones are highly conserved proteins in eukaryotic cells, which can associate with DNA to make up the nucleosomes, thereby constituting the basic unit of chromatin structure. Histones are modified by a series of specific enzymes, and the types of modifications include methylation, acetylation, phosphorylation, and ubiquitination [63]. Moreover, histone modification functions as a crucial part of the regulation of transcription, DNA replication, DNA repairment and chromatin aggregation. Current studies on histone modification mainly focus on the specific amino acid residues of histone H3 and H4. At present, many studies are carried out on histone methylation-demethylation, acetylation-deacetylation and lactylation. The following parts will introduce the roles of these aspects in DN.

### Methylation and demethylation

Histone methylation can induce activation or repression of the transcription process, depending on the changes in the number and position of methyl groups caused by the combined action of histone methyltransferase and histone demethylase. In the glomerular mesangial cells of the diabetic model, H3 histone modification plays an important role. H3 histone lysine methylation (H3Kme) can augment the expressions of TGF-β1 and the extracellular matrix (ECM) proteins-related genes, such as *CTGF*, *collagen-α1* and *PAI-1* [ 64, 65]. Moreover, H3K4me1/2/3, H3K36me2/3, and H3K79me2 can accumulate the pro-inflammatory cytokines and exacerbate the ECM during glomerular fibrosis, ultimately leading to the deterioration of DN [66]. Conversely, H3K9me2/3 and H3K27me3 can inhibit the expressions of fibrotic factors, superoxide dismutase and pro-inflammatory genes, thus decreasing inflammation and fibrosis in the kidney, and eventually delaying the progression of DN [67]. Further experiments demonstrated that the specific histone methyltransferase for H3K27me is the enhancer of zeste homolog 2 (Ezh2), which can accelerate the process of renal fibrosis [68]. H3K27me3 and Ezh2 repress and maintain the expressions of fibrosis and inflammatory genes in the renal mesangial cells under normal conditions [67]. However, this combination in podocytes is suppressed under a diabetic environment [ 69, 70], and TGF-β inhibits the H3K27me3 and Ezh2 levels to mediate mesangial dysfunction, thus eventually leading to kidney damage [67]. In addition, Ezh2-methylated H3K27 can serve as an anchor point for CpG methylation, leading to the formation of silent chromatin, and ultimately, to transcriptional gene silencing [71]. Therefore, further investigation is necessary to clarify the role of Ezh2 in the pathological process of DN development.

In contrast, H3K27me-specific histone demethylases are lysine-specific demethylase 6A (KDM6A, also known as UTX) and 6B [72]. Previous studies have pointed out that the expression of KDM6A increases in the kidney tissue of diabetic mice and DN patients [73]. Moreover, the use of KDM6A inhibitor or si-RNA in diabetic model mice improves renal dysfunction [73]. In addition, pathophysiological indicators, such as proteinuria level, kidney weight, apoptosis, thickening of GBM, and fibrosis are improved in KDM6A-knockout diabetic mice [74]. Meanwhile, kruppel-like factor, as a transcription factor, can enhance the dedifferentiation effect of KDM6A on podocytes, leading to the disappearance of foot processes [74], which deserve further investigation to find the potential therapy targets of DN.

Histone methyltransferase plays an important role in hyperglycemia-mediated injury as well. Previous studies showed that SET7/9 is related to the regulation of the TGF-β1/p21 pathway in chronic kidney disease [65]. In our previous experiment, SET8 was found to be a member of the SET domain-containing methyltransferase family, which is involved in the production of hyperglycemic memory [75]. SET8, as the only known lysine methyltransferase involved in the monomethylation of lysine 20 of histone H4 (H4K20) [76], modulates the expressions of proinflammatory enzymes and NLRP3 inflammasome activation in the hyperglycemic HUVECs [ 77– 79]. Furthermore, overexpression of SET8 leads to histone methylation, thereby regulating downstream signaling pathways and protecting DN [80].

### Acetylation and deacetylation

Over the decades, the research about acetylation and deacetylation of histone in T2DM and microvascular complications has increased gradually. This key process is catalyzed by histone acetyltransferase (HAT) and histone deacetylase (HDAC). Among them, acetylation loosens chromosomal DNA and activates gene expression, while histone deacetylase inhibits gene transcription [81].

HAT catalyzes the acetylation of histones, relaxes the chromatin structure, and promotes transcription positively. In DN, the hyperglycemic environment increases the activities and expression levels of HATs, including p300, CREB-binding protein (CBP), and CBP-associated factor (CAF) [82]. In addition to histones, HAT also acetylates a variety of other proteins such as S-mads, p53/Sp1, and NF-κB, which would further mediate the upregulation of pro-inflammatory cytokines and ECM, and deteriorate the process of DN fibrosis [83]. As an activator, CAF possesses the activity of an intrinsic HAT [84]. Some studies further proved that CAF is closely correlated with the H3K18Ac levels, and enriched on the promoters of inflammatory molecules ICAM-1 and MCP-1. Intervention at this site has the potential to improve inflammation-related renal diseases [85]. Additionally, CBP and p300, as transcriptional co-activators of many vital transcription factors, can play a vital regulatory role in epigenetics by catalyzing the acetylation of histones and transcription factors [86]. Because of their similar structure and redundant function, CBP and p300 often refer to combine jointly. In renal mesangial cells, TGF-induced PAI-1 and p21 are highly related to the interaction between p300/CBP and Smads or Sp1, while H3K9/14Ac increases the p300/CBP-induced promoter activation, which in turn exacerbates the glomerular dysfunction associated with DN [87]. The subsequent experiments demonstrated that curcumin analogue, C66, as an inhibitor of CBP/p300, can protect renal injury in diabetic mice via restraining the expression of JNK and inhibiting the diabetes-related increase in the expression of p300/CBP and acetylation of H3K9/14Ac [88]. In addition, C66 can protect diabetic aortic pathological changes by inhibiting JNK function, accompanied by a boost in the Nrf2 expression [89]. In summary, these data implied that HAT plays a critical role in acetylating histones and may be a potential target for the treatment of DN.

As mentioned above, histone deacetylase inhibits gene transcription in the process of histone acetylation and deacetylation. Till now, 18 HDACs have been found and classified into 4 distinct classes according to their homology to yeast HDAC, including Class I (HDACs1, 2, 3, and 8); Class II being composed of II-a (HDACs4, 5, 7, and 9) and II-b (HDACs6 and 10); Class III, sirtuins (SIRTs1–7) and Class IV (HDAC11) [90]. Different HDACs participate in the pathogenesis of DN through distinct pathways. Among the 4 different classes, HDAC1, as a pro-apoptotic factor, participates in TGF-β1-induced apoptosis [91]. HDAC2 can promote fibrosis [92]. In TGF-β1-treated cells, the knockdown of HDAC2 can reduce the ECM components, implicating the impact of HDAC2 on fibrosis in the kidney [93]. Hydrogen peroxide (H _2_O _2_), known as a potent oxidative stressor, also increases the level of HDAC2 [93], and this could be the latent mechanism of DN which deserves further study. HDAC4 was confirmed as a contributor to podocyte damage in diabetic patients, and it can inhibit autophagy through deacetylation of STAT1 [94]. In addition, sirtuins are also involved in the development of diabetes. SIRT1 inhibits high glucose-induced senescence of vascular cells by reducing ROS accumulation [95]. p66Shc is mainly expressed in renal tubular cells, and a previous study showed that high glucose-induced down-regulation of sirtuin-1 promotes p66Shc expression by increasing the levels of histone H3 and p66Shc acetylation [96]. Normally in glomerular mesangial cells, SIRT1 not only intercepts the activity of the pro-hypertrophic Akt signaling pathway, but also enhances the anti-hypertrophic AMP-activated protein kinase (AMPK) activation [97]. However, the high glucose-induced decrease of SIRT1 leads to the activation of HIF-1α, which induces the expressions of endothelin-1, TGF-β1 and VEGF, thus leading to the pathological angiogenesis and fibrosis of the kidney. It has also been reported that the progress of inflammation and fibrosis can be reversed by mir-217 gene silencing through regulating the SIRT1/HIF-1α signaling pathway [98]. The progress of peritubular capillary rarefaction and fibrosis can be observed in the mice with knockout of SIRT1, and SIRT1 can ameliorate albuminuria actively in diabetic mice [99].

As an inhibitor of HDAC, valproate facilitates autophagy and depresses the NF-κB/iNOS signaling, thereby improving the podocyte damage and renal injuries [100]. Treatment with sodium butyrate (NaB) can significantly decrease the levels of blood sugar and creatinine, improve histological alterations including collagen accumulation and fibrosis, and inhibit the expressions of HDACs, NF-κB activation, and DNA damage in the diabetic kidney tissue [101]. NaB can also upregulate the expression of Nrf2. Compared with the wild-type mice treated with NaB, C57BL/6 Nrf2-knockout mice developed more severe oxidative stress and inflammation in the aortic endothelium [102]. Moreover, NaB increases the synergistic effect between the transcription factor and the p300 on the promoter of the Nrf2, which could be abolished by the p300 inhibitor C646 [102].

### Lactylation

With the in-depth research on histone, a new type of histone modification, named histone lactylation, was identified [103]. Lactylation of histone lysine residues serves as an epigenetic modification that directly stimulates gene transcription [104]. In a previous study, histone lactylation was found to accelerate the pulmonary fibrosis of mice and humans [105]. This modification pattern also promotes tumorigenesis through activating m ^6^A reader protein directly [106]. In addition, a histone modification and transcription assay revealed that histone lactylation can directly activate gene transcription in a p53-dependent p300-mediated pathway [104], it means that p300 is not only an acetyltransferase, but also a promising candidate lactyltransferase. Currently, histone lactylation has not been reported in DN. Therefore, this new mode of histone modification is worthy of further study.

## Non-coding RNAs

Recent studies have demonstrated that non-coding RNAs (ncRNAs) play a vital role in the progression of renal disease, and may be adopted as novel biomarkers and treatment sites of the DN. ncRNA refers to RNA that does not encode any protein, and the most famous ones are miRNAs and long non-coding RNAs [ 32, 107, 108]. They regulate the expressions of genes through modulating protein synthesis at the post-transcription and translation levels [109].

miRNAs, as a kind of endogenous ncRNAs composed of 22 nucleotides, can bear the function of degrading or inhibiting translation through participating in the regulation of post-transcriptional gene expressions via binding to target mRNA [110]. Most of them are the primitive transcripts produced by RNA polymerase acting on the intron regions of protein-coding genes [111]. Researchers have illustrated that the process of miRNA regulation is complex, which means that a single miRNA can simultaneously regulate multiple target genes and vice versa [ 112, 113]. What’s more, numerous pieces of evidence have shown that the expression, regulation, and localization of miRNA can be modulated by changes in the cellular environment accordingly [114].

The hyperglycemia environment can increase the expressions of part of the miRNAs. Among them, miR-21 expression is elevated in DN by inhibiting the expression of TIMP3. As a result, high glucose-induced inflammatory responses and podocyte apoptosis are aggravated in the DN patients [115]. The other mechanism by which miR-21 regulates renal injury is via the regulation of Smad7 level. Knockdown of miR-21 would restrain the TGF-β and NF-κB signaling pathways and restore Smad7 level in diabetic mice [116]. Moreover, it has been shown that miR-21 can enhance the leakage of the slit membrane through inhibiting PTEN-mediated movement of podocytes, thus leading to albuminuria [117]. At the same time, the changing trend of miR-21 in serum is consistent with that in kidney tissue, which enables the level of miR-21 in serum to reflect the kidney function indirectly and to be regarded as a biomarker for the diagnosis of DN [118]. Another oxidative stress-related miRNA is miR-217, and it was characterized in podocytes cultured in a hyperglycemic environment. PTEN is the target of miR-217, which would affect cell apoptosis and ROS production through the pathway of PI3K/AKT/mTOR [119]. From the aspect of inflammation, miR-27 as a pro-inflammatory miRNA, can negatively regulate Nrf2 [120] and PPARγ/β-catenin [121], thus inducing the pro-inflammatory cytokines in the podocytes of the diabetic model. EMT, carried out by the EMT-activated transcription factor (EMT-TF), is one of the important mechanisms of tissue fibrosis. It has been demonstrated that the ubiquitin E3 ligase complex Skp1-Pam-Fbxo45 (SPFFbxo45) can dynamically repress EMT-TF, while miR-27a can reduce the expression of Fbxo45 directly, thereby hindering the degradation of EMT-TF and guaranteeing the occurrence of EMT [122]. Different from the aforementioned findings, another study indicated a nephroprotective role of miRNA-29 [123]. Wang *et al*. [123] discovered low levels of miRNA-29 exist in high glucose-induced early-stage renal fibrosis, advanced diabetic renal fibrosis, and advanced nondiabetic kidney disease. However, another study reported that the expression of miR-29c was higher in kidney tissue, urine sediment, and blood samples of DN patients than that in normal controls. Finally, in-depth research demonstrated that miR-29c is closely related to the increased secretion of inflammatory cytokines [124]. In HK-2 cells cultured in high glucose medium, miR-34a-5p accelerates the transcriptions of SIRT1-related fibrotic genes through the signaling of SIRT1/TGF-β [125]. Furthermore, miR-133b and miR-199b can also induce EMT and renal fibrosis through SIRT1/TGF-β pathway [126].

Among the down-regulated miRNAs, miR-192 is dysregulated and mediates the activation of TGF-β/Smad3 signaling in the early development of renal fibrosis [ 127, 128]. In the kidneys of diabetic rats, TGF-β targets ZEB1/2 in the proximal tubular epithelial cells through silencing miR-192 expression. It should be noted that ZEB1 and ZEB2 are E-box-binding proteins which have an important impact on the early phase of EMT [129]. In addition, the miR-192 level in serum and urine is relatively stable and hard to degrade, which may denote that miR-192 could be used to diagnose the level of kidney damage better than the commonly used clinical test index, i.e., the albumin-to-creatinine ratio [130]. Another downregulated miRNA in DN is miR-30e, and the overexpression of miR-30e can promote the proliferation of renal tubular endothelial cells and inhibit EMT through inhibiting GLIPR-2 expression, thus ultimately avoiding renal fibrosis [131]. Moreover, miR-25 decreases in a time-dependent manner in HK-2 cells cultured in high glucose medium. Overexpression of miR-25 can inhibit the production of ROS and activate the PTEN/AKT pathway to produce anti-apoptotic effects [132]. Liu *et al*. [133] also found that miR-25 overexpression in the podocytes of DN mice can reduce proteinuria, attenuate glomerular fibrosis, and inhibit the RAS system to decrease renal hypertension. Furthermore, monocyte chemoattractant protein (MCP)-1 recruits macrophages to inflammatory sites, thus aggravating the development and progression of DN. It was reported that miR-374a has an anti-inflammatory effect through the negative regulation of MCP-1 expression [134]. In DN patients, restoration of miR-374a expression can effectively prevent inflammation in renal tubular epithelial cells [134].

## Long Non-coding RNAs

As mentioned above, long non-coding RNAs (lncRNAs), which have a nucleotide length greater than 200 nt with no protein-coding function, is another type of ncRNAs that can regulate gene expression. Similar to protein-coding mRNAs, lncRNA is also transcribed by RNA polymerase II or polymerase III, and most of them have 5-caps and 3-terminal polytails. However, the conserved sequences between species are less than 10%, and the expression abundance is not high. Therefore, they have strong tissue and cell specificity [ 135– 137]. In terms of its function, lncRNA can regulate gene expression at the levels of transcription, post-transcription, and translation [138]. The biological evidence for extracellular lncRNA is limited, but numerous studies have successfully illustrated that, similar to miRNA, lncRNAs exist in vesicles or circulate freely in biological fluids [139]. More importantly, under normal circumstances, lncRNAs are usually expressed at low levels, but the levels will increase specifically at a specific stage of disease progression [ 140, 141].

As the first lncRNA that has been demonstrated to be related to kidney diseases, plasmacytoma variant translocation (PVT1) was confirmed to have a close relationship with the occurrence and development of DN [142]. As a type of lncRNA located in the 8q24 region of the human chromosome, the increase of PVT1 expression would promote cell proliferation and inhibit cell apoptosis [143]. In addition, the expression of PVT1 was found to be upregulated in glomerular mesangial cells under high glucose conditions. Meanwhile, PVT1 regulates the expression of the main components of ECM and its main regulator PAI-1 in a manner independent of the TGF-β1 pathway. Compared with the effect of inhibiting TGF-β1, knockout of *PVT1* was proved to be a more effective approach in reducing the levels of FN1, COL4A1, and PAI-1 [144]. Moreover, it has been shown that under high glucose environment both PVT1 and its derivative miR-1207-5p can enhance the expressions of FN1, TGF-β1, and PAI-1 in glomerular mesangial cells independently of each other, thereby increasing ECM accumulation and accelerating the process of renal fibrosis of DN [145]. In addition, PVT1 also activates the pathway of PI3K/Akt/mTOR via up-regulating miR-93-5p [146], which promotes the progress of cell proliferation, migration and invasion. Apart from PVT1, H19 is also expressed in the nucleus and is significantly increased in some diseased conditions [ 147, 148], which has been reported to participate in renal diseases [149]. It has been reported that H19 is increased in TGF-β2-induced fibrosis in proximal tubular cells [150], implying that inhibition of H19 alleviates fibrosis and reconstructs normal tissue of the kidney. Meanwhile, it is interesting to note that inhibition of H19 also changes miR-29a level and inhibits endothelial-to-mesenchymal transition (EndMT) through attenuating the TGF-β/Smad signaling pathway, leading to the block of fibrosis in DN [151].

Hyperglycemia can promote inflammation, oxidative stress, and fibrosis of the kidneys and it is an important cause of the occurrence and development of DN. Among these three adverse consequences, chronic inflammation plays a particularly important role in the early stage of DN. It was reported that knockout of lncRNA Gm4419 can improve NF-κB/NLRP3 inflammatory complex-mediated cell inflammation, fibrosis, and proliferation [152]. Similarly, increased expression of lncRNA GM6135 promotes inflammatory reaction by augmenting TLR4 expression in diabetic mice. As a member of TLR-mediated signaling, TLR4 secrets pro-inflammatory cytokines by sponging related miRNAs [153]. In addition, lncRNA Tug1 not only participates in regulating ECM accumulation, but also regulates the process of DN via modulating mitochondrial damage. Meanwhile, it has been proven that PGC-1α plays an important role in cell energy and mitochondrial homeostasis, while the expression levels of PGC-1α and lncRNA Tug1 were decreased in diabetic environment [154]. Similarly, Long *et al*. [155] denoted that overexpression of lncRNA Tug1 can augment the expression of PGC-1α and repair mitochondrial damage. Moreover, Tug1 can promote PGC-1α expression and improve mitochondrial energy balance, thus delaying the progression of DN [155]. In addition, Tug1 antagonizs the effect of miR-377 on downregulating PPARγ, and inhibits high glucose-mediated ECM accumulation. Meanwhile, lncRNA TUG1, as a response of miR-377, can reduce miR-377 expression, thereby inhibiting its target gene *PPARγ* and alleviating the accumulation of ECM in renal mesangial cells [156]. Apart from that, the level of lncRNA MIAT is increased in retinal endothelial cells under a high glucose environment, while knockout of MIAT can significantly improve diabetic retinal microangiopathy and inhibit the proliferation, migration, and blood vessel formation of retinal endothelial cells. As a competitive endogenous RNA, MIAT can regulate endothelial cell function by forming a regulatory pathway together with VEGF and miRNA-150-5p, thus participating in diabetic microangiopathy [157]. Simultaneously, the level of lncRNA MIAT in the kidney tissue of diabetic mice is also decreased, and it is negatively correlated with the creatinine and urea nitrogen levels. In the meantime, overexpression of MIAT can reverse the inhibitory effect of high glucose on Nrf2 expression, confirming that MIAT can regulate the viability of HK-2 cells by stabilizing Nrf2 expression and improving the prognosis of DN obviously [158].

## Conclusions

Epigenetics is very important for the pathogenesis and the development of DN. In the past decades, with the development of medical technologies, great progresses have been made in the diagnosis and treatment of DN. Among these new technologies and concepts, the epigenetic mechanisms have pioneered novel horizons for the cause of diseases and made great contributions to the development of more meaningful and effective treatments. However, the current knowledge of epigenetics is still quite limited, which means that there are still many unknowns in this field worth further research, especially the novel functions of histone lactylation in DN. In short, actively exploring the unknown aspects of epigenetics is particularly important for reducing the severity and related risks of DN and improving the prognosis of diabetic patients.

## References

[1] Yang W, Lu J, Weng J, Jia W, Ji L, Xiao J, Shan Z (2010). Prevalence of diabetes among men and women in China. N Engl J Med.

[2] Ma Q, Li Y, Li P, Wang M, Wang J, Tang Z, Wang T (2019). Research progress in the relationship between type 2 diabetes mellitus and intestinal flora. Biomed Pharmacother.

[3] Effect of intensive diabetes treatment on the development and progression of long-term complications in adolescents with insulin-dependent diabetes mellitus: Diabetes Control and Complications Trial. Diabetes Control and Complications Trial Research Group.
*
J Pediatr
* 1994, 125: 177-188. https://doi.org/10.1016/s0022-3476(94)70190-3.

[4] The Writing Team for the Diabetes Control and Complications Trial/Epidemiology of Diabetes Interventions and Complications Research Group (2002). Effect of intensive therapy on the microvascular complications of type 1 diabetes mellitus. JAMA.

[5] Writing Team for the Diabetes C, Complications Trial/Epidemiology of Diabetes I, Complications Research G. Sustained effect of intensive treatment of type 1 diabetes mellitus on development and progression of diabetic nephropathy: the Epidemiology of Diabetes Interventions and Complications (EDIC) study.
*
JAMA
* 2003, 290: 2159-2167. https://doi.org/10.1001/jama.290.16.2159.

[6] Patel A, MacMahon S, Chalmers J, Neal B, Billot L, Woodward M, Marre M (2008). Intensive blood glucose control and vascular outcomes in patients with type 2 diabetes. N Engl J Med.

[7] Gerstein HC, Miller ME, Byington RP, Goff DC, Bigger JT, Buse JB, Cushman WC (2008). Effects of intensive glucose lowering in type 2 diabetes. N Engl J Med.

[8] Duckworth W, Abraira C, Moritz T, Reda D, Emanuele N, Reaven PD, Zieve FJ (2009). Glucose control and vascular complications in veterans with type 2 diabetes. N Engl J Med.

[9] Boussageon R, Bejan-Angoulvant T, Saadatian-Elahi M, Lafont S, Bergeonneau C, Kassaï B, Erpeldinger S (2011). Effect of intensive glucose lowering treatment on all cause mortality, cardiovascular death, and microvascular events in type 2 diabetes: meta-analysis of randomised controlled trials. BMJ.

[10] El-Osta A (2012). Glycemic memory. Curr Opin Lipidol.

[11] Ziyadeh FN, Sharma K (2003). Overview: combating diabetic nephropathy. J Am Soc Nephrol.

[12] Natarajan R, Nadler JL (2004). Lipid inflammatory mediators in diabetic vascular disease. Arterioscler Thromb Vasc Biol.

[13] Brownlee M (2005). The pathobiology of diabetic complications: a unifying mechanism. Diabetes.

[14] Zeadin MG, Petlura CI, Werstuck GH (2013). Molecular mechanisms linking diabetes to the accelerated development of atherosclerosis. Canadian J Diabetes.

[15] Geraldes P, King GL (2010). Activation of protein kinase C isoforms and its impact on diabetic complications. Circ Res.

[16] Wendt T, Bucciarelli L, Qu W, Lu Y, Yan SF, Stern DM, Schmidt AM (2002). Receptor for advanced glycation endproducts (RAGE) and vascular inflammation: insights into the pathogenesis of macrovascular complications in diabetes. Curr Atheroscler Rep.

[17] Reddy MA, Li SL, Sahar S, Kim YS, Xu ZG, Lanting L, Natarajan R (2006). Key role of Src kinase in S100B-induced activation of the receptor for advanced glycation end products in vascular smooth muscle cells. J Biol Chem.

[18] Yan SF, Ramasamy R, Schmidt AM (2008). Mechanisms of disease: advanced glycation end-products and their receptor in inflammation and diabetes complications. Nat Rev Endocrinol.

[19] Piqueras L, Sanz MJ (2020). Angiotensin II and leukocyte trafficking: new insights for an old vascular mediator. Role of redox-signaling pathways. Free Radical Biol Med.

[20] Brownlee M (2001). Biochemistry and molecular cell biology of diabetic complications. Nature.

[21] Meng XM, Nikolic-Paterson DJ, Lan HY (2016). TGF-β: the master regulator of fibrosis. Nat Rev Nephrol.

[22] Prattichizzo F, Giuliani A, De Nigris V, Pujadas G, Ceka A, La Sala L, Genovese S (2016). Extracellular microRNAs and endothelial hyperglycaemic memory: a therapeutic opportunity?. Diabetes Obes Metab.

[23] El-Osta A, Brasacchio D, Yao D, Pocai A, Jones PL, Roeder RG, Cooper ME (2008). Transient high glucose causes persistent epigenetic changes and altered gene expression during subsequent normoglycemia. J Exp Med.

[24] Harvey ZH, Chen Y, Jarosz DF (2018). Protein-based inheritance: epigenetics beyond the chromosome. Mol Cell.

[25] Brasacchio D, Okabe J, Tikellis C, Balcerczyk A, George P, Baker EK, Calkin AC (2009). Hyperglycemia induces a dynamic cooperativity of histone methylase and demethylase enzymes associated with gene-activating epigenetic marks that coexist on the lysine tail. Diabetes.

[26] Miao F, Gonzalo IG, Lanting L, Natarajan R (2004). *In vivo* chromatin remodeling events leading to inflammatory gene transcription under diabetic conditions. J Biol Chem.

[27] Kato M, Natarajan R (2019). Epigenetics and epigenomics in diabetic kidney disease and metabolic memory. Nat Rev Nephrol.

[28] Sankrityayan H, Kulkarni YA, Gaikwad AB (2019). Diabetic nephropathy: The regulatory interplay between epigenetics and microRNAs. Pharmacol Res.

[29] Selbach M, Schwanhäusser B, Thierfelder N, Fang Z, Khanin R, Rajewsky N (2008). Widespread changes in protein synthesis induced by microRNAs. Nature.

[30] Testa R, Bonfigli AR, Prattichizzo F, La Sala L, De Nigris V, Ceriello A (2017). The “metabolic memory” theory and the early treatment of hyperglycemia in prevention of diabetic complications. Nutrients.

[31] Harlan SM, Heinz-Taheny KM, Sullivan JM, Wei T, Baker HE, Jaqua DL, Qi Z (2018). Progressive renal disease established by renin-coding adeno-associated virus–driven hypertension in diverse diabetic models. J Am Soc Nephrol.

[32] Dewanjee S, Bhattacharjee N (2018). MicroRNA: a new generation therapeutic target in diabetic nephropathy. Biochem Pharmacol.

[33] Prattichizzo F, Giuliani A, Ceka A, Rippo MR, Bonfigli AR, Testa R, Procopio AD (2015). Epigenetic mechanisms of endothelial dysfunction in type 2 diabetes. Clin Epigenet.

[34] Cantone I, Fisher AG (2013). Epigenetic programming and reprogramming during development. Nat Struct Mol Biol.

[35] Angeloni A, Bogdanovic O (2019). Enhancer DNA methylation: implications for gene regulation. Essays Biochem.

[36] Ehrlich M (2019). DNA hypermethylation in disease: mechanisms and clinical relevance. Epigenetics.

[37] VanderJagt TA, Neugebauer MH, Morgan M, Bowden DW, Shah VO (2015). Epigenetic profiles of pre-diabetes transitioning to type 2 diabetes and nephropathy. World J Diabetes.

[38] Chen Z, Miao F, Paterson AD, Lachin JM, Zhang L, Schones DE, Wu X (2016). Epigenomic profiling reveals an association between persistence of DNA methylation and metabolic memory in the DCCT/EDIC type 1 diabetes cohort. Proc Natl Acad Sci U S A.

[39] Jha JC, Banal C, Chow BSM, Cooper ME, Jandeleit-Dahm K (2016). Diabetes and kidney disease: role of oxidative stress. Antioxid Redox Signal.

[40] Niu Y, DesMarais TL, Tong Z, Yao Y, Costa M (2015). Oxidative stress alters global histone modification and DNA methylation. Free Radical Biol Med.

[41] Zhang YW, Wang Z, Xie W, Cai Y, Xia L, Easwaran H, Luo J (2017). Acetylation enhances TET2 function in protecting against abnormal DNA methylation during oxidative stress. Mol Cell.

[42] Giacco F, Brownlee M (2010). Oxidative stress and diabetic complications. Circ Res.

[43] Paneni F, Mocharla P, Akhmedov A, Costantino S, Osto E, Volpe M, Lüscher TF (2012). Gene silencing of the mitochondrial adaptor p66(Shc) suppresses vascular hyperglycemic memory in diabetes. Circ Res.

[44] Babu M, Durga Devi T, Mäkinen P, Kaikkonen M, Lesch HP, Junttila S, Laiho A (2015). Differential promoter methylation of macrophage genes is associated with impaired vascular growth in ischemic muscles of hyperlipidemic and type 2 diabetic mice. Circ Res.

[45] Wu D, Dasgupta A, Read AD, Bentley RE, Motamed M, Chen KH, Al-Qazazi R,
*et al*. Oxygen sensing, mitochondrial biology and experimental therapeutics for pulmonary hypertension and cancer.
*
Free Radic Biol Med
* 2021, 12: S0891-S5849. https://doi.org/10.1016/j.freeradbiomed.2020.12.452.

[46] Jiang N, Zhao H, Han Y, Li L, Xiong S, Zeng L, Xiao Y (2020). HIF‐1α ameliorates tubular injury in diabetic nephropathy via HO‐1–mediated control of mitochondrial dynamics. Cell Prolif.

[47] Kirito K, Hu Y, Komatsu N (2009). HIF-1 prevents the overproduction of mitochondrial ROS after cytokine stimulation through induction of PDK-1. Cell Cycle.

[48] Zhang L, Zhang Q, Liu S, Chen Y, Li R, Lin T, Yu C (2017). DNA methyltransferase 1 may be a therapy target for attenuating diabetic nephropathy and podocyte injury. Kidney Int.

[49] Sharma I, Dutta RK, Singh NK, Kanwar YS (2017). High glucose–induced hypomethylation promotes binding of Sp-1 to myo-inositol oxygenase. Am J Pathol.

[50] Li Z, Chen H, Zhong F, Zhang W, Lee K, He JC (2019). Expression of glutamate receptor subtype 3 is epigenetically regulated in podocytes under diabetic conditions. Kidney Dis.

[51] Ling L, Chen L, Zhang C, Gui S, Zhao H, Li Z (2018). Highglucose induces podocyteepithelial‑to‑mesenchymal transition by demethylation‑mediated enhancement of mmp9 expression. Mol Med Rep.

[52] Yang XH, Feng SY, Yu Y, Liang Z (2018). Badanie zależności między metylacją regionu promotora genu MMP-9 a nefropatią cukrzycową. Endokrynologia Polska.

[53] Nan X, Ng HH, Johnson CA, Laherty CD, Turner BM, Eisenman RN, Bird A (1998). Transcriptional repression by the methyl-CpG-binding protein MeCP2 involves a histone deacetylase complex. Nature.

[54] Jones PL, Veenstra GJC, Wade PA, Vermaak D, Kass SU, Landsberger N, Strouboulis J (1998). Methylated DNA and MeCP2 recruit histone deacetylase to repress transcription. Nat Genet.

[55] Estève PO, Chin HG, Smallwood A, Feehery GR, Gangisetty O, Karpf AR, Carey MF, Pradhan S. Direct interaction between DNMT1 and G9a coordinates DNA and histone methylation during replication.
*
Genes Dev
*2006, 20: 3089-3103. https://doi.org/10.1101/gad.1463706.

[56] Park J, Guan Y, Sheng X, Gluck C, Seasock MJ, Hakimi AA, Qiu C (2019). Functional methylome analysis of human diabetic kidney disease. JCI Insight.

[57] Idriss HT, Naismith JH (2000). TNF? and the TNF receptor superfamily: Structure-function relationship(s). Microsc Res Tech.

[58] Chen G, Chen H, Ren S, Xia M, Zhu J, Liu Y, Zhang L (2019). Aberrant DNA methylation of mTOR pathway genes promotes inflammatory activation of immune cells in diabetic kidney disease. Kidney Int.

[59] Qiu C, Hanson RL, Fufaa G, Kobes S, Gluck C, Huang J, Chen Y (2018). Cytosine methylation predicts renal function decline in American Indians. Kidney Int.

[60] Maier M, Baldwin C, Aoudjit L, Takano T (2018). The role of trio, a rho guanine nucleotide exchange factor, in glomerular podocytes. Int J Mol Sci.

[61] Annett S, Moore G, Short A, Marshall A, McCrudden C, Yakkundi A, Das S (2020). FKBPL-based peptide, ALM201, targets angiogenesis and cancer stem cells in ovarian cancer. Br J Cancer.

[62] Thuerauf DJ, Marcinko M, Belmont PJ, Glembotski CC (2007). Effects of the isoform-specific characteristics of ATF6α and ATF6β on endoplasmic reticulum stress response gene expression and cell viability. J Biol Chem.

[63] Emamgholipour S, Ebrahimi R, Bahiraee A, Niazpour F, Meshkani R (2020). Acetylation and insulin resistance: a focus on metabolic and mitogenic cascades of insulin signaling. Crit Rev Clin Laboratory Sci.

[64] Tsuchida KI, Zhu Y, Siva S, Dunn SR, Sharma K (2003). Role of Smad4 on TGF-β–induced extracellular matrix stimulation in mesangial cells. Kidney Int.

[65] Guo Q, Li X, Han H, Li C, Liu S, Gao W, Sun G (2016). Histone lysine methylation in TGF-
*β*1 mediated p21 gene expression in rat mesangial cells. Biomed Res Int.

[66] Sun G, Cui W, Guo Q, Miao L (2014). Histone lysine methylation in diabetic nephropathy. J Diabetes Res.

[67] Jia Y, Reddy MA, Das S, Oh HJ, Abdollahi M, Yuan H, Zhang E (2019). Dysregulation of histone H3 lysine 27 trimethylation in transforming growth factor-β1–induced gene expression in mesangial cells and diabetic kidney. J Biol Chem.

[68] Zhou X, Zang X, Ponnusamy M, Masucci MV, Tolbert E, Gong R, Zhao TC (2016). Enhancer of zeste homolog 2 inhibition attenuates renal fibrosis by maintaining Smad7 and phosphatase and tensin homolog expression. J Am Soc Nephrol.

[69] Majumder S, Thieme K, Batchu SN, Alghamdi TA, Bowskill BB, Kabir MG, Liu Y (2018). Shifts in podocyte histone H3K27me3 regulate mouse and human glomerular disease. J Clin Investigation.

[70] Siddiqi FS, Majumder S, Thai K, Abdalla M, Hu P, Advani SL, White KE (2016). The histone methyltransferase enzyme enhancer of zeste homolog 2 protects against podocyte oxidative stress and renal injury in diabetes. J Am Soc Nephrol.

[71] Wei JW, Huang K, Yang C, Kang CS (2017). Non-coding RNAs as regulators in epigenetics. Oncol Rep.

[72] Agger K, Cloos PAC, Christensen J, Pasini D, Rose S, Rappsilber J, Issaeva I (2007). UTX and JMJD3 are histone H3K27 demethylases involved in HOX gene regulation and development. Nature.

[73] Chen H, Huang Y, Zhu X, Liu C, Yuan Y, Su H, Zhang C (2019). Histone demethylase UTX is a therapeutic target for diabetic kidney disease. J Physiol.

[74] Lin CL, Hsu YC, Huang YT, Shih YH, Wang CJ, Chiang WC, Chang PJ (2019). A KDM6A–KLF10 reinforcing feedback mechanism aggravates diabetic podocyte dysfunction. EMBO Mol Med.

[75] Chen X, Wu Q, Jiang H, Wang J, Zhao Y, Xu Y, Zhu M (2018). SET8 is involved in the regulation of hyperglycemic memory in human umbilical endothelial cells. Acta Biochim Biophys Sin.

[76] Fang J, Feng Q, Ketel CS, Wang H, Cao R, Xia L, Erdjument-Bromage H (2002). Purification and functional characterization of SET8, a nucleosomal histone H4-lysine 20-specific methyltransferase. Curr Biol.

[77] Qi J, Wu Q, Cheng Q, Chen X, Zhu M, Miao C (2020). High glucose induces endothelial COX2 and iNOS expression via inhibition of monomethyltransferase SETD8 expression. J Diabetes Res.

[78] Wang J, Shen X, Liu J, Chen W, Wu F, Wu W, Meng Z (2020). High glucose mediates NLRP3 inflammasome activation via upregulation of ELF3 expression. Cell Death Dis.

[79] Shen X, Chen X, Wang J, Liu J, Wang Z, Hua Q, Wu Q (2020). SET8 suppression mediates high glucose-induced vascular endothelial inflammation via the upregulation of PTEN. Exp Mol Med.

[80] Huang T, Li X, Wang F, Lu L, Hou W, Zhu M, Miao C (2021). The CREB/KMT5A complex regulates PTP1B to modulate high glucose-induced endothelial inflammatory factor levels in diabetic nephropathy. Cell Death Dis.

[81] Sharma S, Taliyan R (2016). Histone deacetylase inhibitors: future therapeutics for insulin resistance and type 2 diabetes. Pharmacol Res.

[82] Li X, Li C, Sun G (2016). Histone acetylation and its modifiers in the pathogenesis of diabetic nephropathy. J Diabetes Res.

[83] Nie L, Liu Y, Zhang B, Zhao J (2020). Application of histone deacetylase inhibitors in renal interstitial fibrosis. Kidney Dis.

[84] Soutoglou E, Viollet B, Vaxillaire M, Yaniv M, Pontoglio M, Talianidis I (2001). Transcription factor-dependent regulation of CBP and P/CAF histone acetyltransferase activity. EMBO J.

[85] Huang J, Wan D, Li J, Chen H, Huang K, Zheng L (2015). Histone acetyltransferase PCAF regulates inflammatory molecules in the development of renal injury. Epigenetics.

[86] Ghosh AK, Varga J (2007). The transcriptional coactivator and acetyltransferase p300 in fibroblast biology and fibrosis. J Cell Physiol.

[87] Yuan H, Reddy MA, Sun G, Lanting L, Wang M, Kato M, Natarajan R (2013). Involvement of p300/CBP and epigenetic histone acetylation in TGF-β1-mediated gene transcription in mesangial cells. Am J Physiol-Renal Physiol.

[88] Wang Y, Wang Y, Luo M, Wu H, Kong L, Xin Y, Cui W (2015). Novel curcumin analog C66 prevents diabetic nephropathy via JNK pathway with the involvement of p300/CBP-mediated histone acetylation. BioChim Biophys Acta (BBA) - Mol Basis Dis.

[89] Liu Y, Wang Y, Miao X, Zhou S, Tan Y, Liang G, Zheng Y (2014). Inhibition of
^JNK^ by compound C66 prevents pathological changes of the aorta in
^STZ^ ‐induced diabetes. J Cell Mol Med.

[90] Hadden MJ, Advani A (2018). Histone deacetylase inhibitors and diabetic kidney disease. Int J Mol Sci.

[91] Lei WW, Zhang KH, Pan XC, Wang DM, Hu Y, Yang YN, Song JG. Histone deacetylase 1 and 2 differentially regulate apoptosis by opposing effects on extracellular signal-regulated kinase 1/2.
*
Cell Death Dis
* 2010, 2041-4889/10. 10.1038/cddis.2010.21.

[92] Zhang L, Chen L, Gao C, Chen E, Lightle AR, Foulke L, Zhao B (2020). Loss of histone H3 K79 methyltransferase Dot1l facilitates kidney fibrosis by upregulating endothelin 1 through histone deacetylase 2. J Am Soc Nephrol.

[93] Noh H, Oh EY, Seo JY, Yu MR, Kim YO, Ha H, Lee HB (2009). Histone deacetylase-2 is a key regulator of diabetes- and transforming growth factor-β1-induced renal injury. Am J Physiol-Renal Physiol.

[94] Wei Q, Dong Z (2014). HDAC4 blocks autophagy to trigger podocyte injury: non-epigenetic action in diabetic nephropathy. Kidney Int.

[95] Chen HZ, Wan YZ, Zhou S, Lu YB, Zhang ZQ, Zhang R, Chen F (2012). Endothelium-specific SIRT1 overexpression inhibits hyperglycemia-induced upregulation of vascular cell senescence. Sci China Life Sci.

[96] Zhou S, Chen HZ, Wan YZ, Zhang QJ, Wei YS, Huang S, Liu JJ (2011). Repression of P66Shc expression by SIRT1 contributes to the prevention of hyperglycemia-induced endothelial dysfunction. Circ Res.

[97] Baek D, Villén J, Shin C, Camargo FD, Gygi SP, Bartel DP (2008). The impact of microRNAs on protein output. Nature.

[98] Shao Y, Lv C, Wu C, Zhou Y, Wang Q (2016). Mir-217 promotes inflammation and fibrosis in high glucose cultured rat glomerular mesangial cells via Sirt1/HIF-1α signaling pathway. Diabetes Metab Res Rev.

[99] Hasegawa K, Wakino S, Simic P, Sakamaki Y, Minakuchi H, Fujimura K, Hosoya K (2013). Renal tubular Sirt1 attenuates diabetic albuminuria by epigenetically suppressing Claudin-1 overexpression in podocytes. Nat Med.

[100] Khan S, Jena G, Tikoo K, Kumar V (2015). Valproate attenuates the proteinuria, podocyte and renal injury by facilitating autophagy and inactivation of NF-κB/iNOS signaling in diabetic rat. Biochimie.

[101] Khan S, Jena G (2014). Sodium butyrate, a HDAC inhibitor ameliorates eNOS, iNOS and TGF-β1-induced fibrogenesis, apoptosis and DNA damage in the kidney of juvenile diabetic rats. Food Chem Toxicol.

[102] Wu J, Jiang Z, Zhang H, Liang W, Huang W, Zhang H, Li Y (2018). Sodium butyrate attenuates diabetes-induced aortic endothelial dysfunction via P300-mediated transcriptional activation of Nrf2. Free Radical Biol Med.

[103] Sabari BR, Zhang D, Allis CD, Zhao Y (2017). Metabolic regulation of gene expression through histone acylations. Nat Rev Mol Cell Biol.

[104] Zhang D, Tang Z, Huang H, Zhou G, Cui C, Weng Y, Liu W (2019). Metabolic regulation of gene expression by histone lactylation. Nature.

[105] Cui H, Xie N, Banerjee S, Ge J, Jiang D, Dey T, Matthews QL (2021). Lung myofibroblasts promote macrophage profibrotic activity through lactate-induced histone lactylation. Am J Respir Cell Mol Biol.

[106] Yu J, Chai P, Xie M, Ge S, Ruan J, Fan X, Jia R (2021). Histone lactylation drives oncogenesis by facilitating m6A reader protein YTHDF2 expression in ocular melanoma. Genome Biol.

[107] Abu N, Jamal R (2016). Circular RNAs as promising biomarkers: a mini-review. Front Physiol.

[108] Alvarez ML, Distefano JK (2013). The role of non-coding RNAs in diabetic nephropathy: potential applications as biomarkers for disease development and progression. Diabetes Res Clin Pract.

[109] Taft RJ, Pang KC, Mercer TR, Dinger M, Mattick JS (2010). Non-coding RNAs: regulators of disease. J Pathol.

[110] Shi Y, Vanhoutte PM (2017). Macro- and microvascular endothelial dysfunction in diabetes. J Diabetes.

[111] Rodriguez A, Griffiths-Jones S, Ashurst JL, Bradley A (2004). Identification of mammalian microRNA host genes and transcription units. Genome Res.

[112] Chowdhury AR, Chetty M, Vinh NX (2014). Evaluating influence of microRNA in reconstructing gene regulatory networks. Cogn Neurodyn.

[113] Towler BP, Jones CI, Newbury SF (2015). Mechanisms of regulation of mature miRNAs. Biochem Soc Trans.

[114] Ebert MS, Sharp PA (2012). Roles for microRNAs in conferring robustness to biological processes. Cell.

[115] Chen X, Zhao L, Xing Y, Lin B (2018). Down-regulation of microRNA-21 reduces inflammation and podocyte apoptosis in diabetic nephropathy by relieving the repression of TIMP3 expression. Biomed Pharmacother.

[116] Zhong X, Chung ACK, Chen HY, Dong Y, Meng XM, Li R, Yang W (2013). miR-21 is a key therapeutic target for renal injury in a mouse model of type 2 diabetes. Diabetologia.

[117] Kölling M, Kaucsar T, Schauerte C, Hübner A, Dettling A, Park JK, Busch M (2017). Therapeutic miR-21 silencing ameliorates diabetic kidney disease in mice. Mol Ther.

[118] Wang J, Duan L, Tian L, Liu J, Wang S, Gao Y, Yang J (2016). Serum miR-21 may be a potential diagnostic biomarker for diabetic nephropathy. Exp Clin Endocrinol Diabetes.

[119] Sun J, Li ZP, Zhang RQ, Zhang HM (2017). Repression of miR-217 protects against high glucose-induced podocyte injury and insulin resistance by restoring PTEN-mediated autophagy pathway. Biochem Biophys Res Commun.

[120] Zhao Y, Dong D, Reece EA, Wang AR, Yang P (2018). Oxidative stress-induced miR-27a targets the redox gene nuclear factor erythroid 2-related factor 2 in diabetic embryopathy. Am J Obstet GynEcol.

[121] Zhou Z, Wan J, Hou X, Geng J, Li X, Bai X (2017). MicroRNA-27a promotes podocyte injury via PPARγ-mediated β-catenin activation in diabetic nephropathy. Cell Death Dis.

[122] Xu M, Zhu C, Zhao X, Chen C, Zhang H, Yuan H, Deng R (2019). Correction: Atypical ubiquitin E3 ligase complex Skp1-Pam-Fbxo45 controls the core epithelial-to-mesenchymal transition-inducing transcription factors. Oncotarget.

[123] Wang B, Komers R, Carew R, Winbanks CE, Xu B, Herman-Edelstein M, Koh P (2012). Suppression of microRNA-29 Expression by TGF-
*β* 1 Promotes Collagen Expression and Renal Fibrosis. J Am Soc Nephrol.

[124] Guo J, Li J, Zhao J, Yang S, Wang L, Cheng G, Liu D (2017). MiRNA-29c regulates the expression of inflammatory cytokines in diabetic nephropathy by targeting tristetraprolin. Sci Rep.

[125] Xue M, Li Y, Hu F, Jia YJ, Zheng ZJ, Wang L, Xue YM (2018). High glucose up-regulates microRNA-34a-5p to aggravate fibrosis by targeting SIRT1 in HK-2 cells. Biochem Biophys Res Commun.

[126] Sun Z, Ma Y, Chen F, Wang S, Chen B, Shi J (2018). miR-133b and miR-199b knockdown attenuate TGF-β1-induced epithelial to mesenchymal transition and renal fibrosis by targeting SIRT1 in diabetic nephropathy. Eur J Pharmacol.

[127] Chung ACK, Huang XR, Meng X, Lan HY (2010). miR-192 mediates TGF-β/Smad3-driven renal fibrosis. J Am Soc Nephrol.

[128] Meng XM, Tang PMK, Li J, Lan HY (2015). TGF-Î²/Smad signaling in renal fibrosis. Front Physiol.

[129] Wang B, Herman-Edelstein M, Koh P, Burns W, Jandeleit-Dahm K, Watson A, Saleem M (2010). E-cadherin expression is regulated by miR-192/215 by a mechanism that is independent of the profibrotic effects of transforming growth factor-beta. Diabetes.

[130] Yang X, Liu S, Zhang R, Sun B, Zhou S, Chen R, Yu P (2017). Microribonucleic acid-192 as a specific biomarker for the early diagnosis of diabetic kidney disease. J Diabetes Investig.

[131] Zhao D, Jia J, Shao H (2017). miR-30e targets GLIPR-2 to modulate diabetic nephropathy:
*in vitro* and
*in vivo* experiments. J Mol Endocrinol.

[132] Li H, Zhu X, Zhang J, Shi J (2017). MicroRNA-25 inhibits high glucose-induced apoptosis in renal tubular epithelial cells via PTEN/AKT pathway. Biomed Pharmacother.

[133] Liu Y, Li H, Liu J, Han P, Li X, Bai H, Zhang C (2017). Variations in microRNA-25 expression influence the severity of diabetic kidney disease. J Am Soc Nephrol.

[134] Yang Z, Guo Z, Dong J, Sheng S, Wang Y, Yu L, Wang H (2018). miR-374a regulates inflammatory response in diabetic nephropathy by targeting MCP-1 expression. Front Pharmacol.

[135] Kapranov P, Cheng J, Dike S, Nix DA, Duttagupta R, Willingham AT, Stadler PF (2007). RNA maps reveal new RNA classes and a possible function for pervasive transcription. Science.

[136] Ignarski M, Islam R, Müller RU (2019). Long non-coding RNAs in kidney disease. Int J Mol Sci.

[137] Quinn JJ, Chang HY (2016). Unique features of long non-coding RNA biogenesis and function. Nat Rev Genet.

[138] Rashid F, Shah A, Shan G (2016). Long non-coding RNAs in the cytoplasm. Genomics Proteomics BioInf.

[139] Pathania AS, Challagundla KB (2021). Exosomal long non-coding RNAs: emerging players in the tumor microenvironment. Mol Ther - Nucleic Acids.

[140] Hung T, Chang HY (2010). Long noncoding RNA in genome regulation. RNA Biol.

[141] Goyal N, Kesharwani D, Datta M (2018). Lnc-ing non-coding RNAs with metabolism and diabetes: roles of lncRNAs. Cell Mol Life Sci.

[142] Hanson RL, Craig DW, Millis MP, Yeatts KA, Kobes S, Pearson JV, Lee AM (2007). Identification of PVT1 as a candidate gene for end-stage renal disease in type 2 diabetes using a pooling-based genome-wide single nucleotide polymorphism association study. Diabetes.

[143] Huppi K, Volfovsky N, Runfola T, Jones TL, Mackiewicz M, Martin SE, Mushinski JF (2008). The identification of microRNAs in a genomically unstable region of human chromosome 8q24. Mol Cancer Res.

[144] Alvarez ML, DiStefano JK (2011). Functional characterization of the plasmacytoma variant translocation 1 gene (PVT1) in diabetic nephropathy. PLoS ONE.

[145] Alvarez ML, Khosroheidari M, Eddy E, Kiefer J (2013). Role of microRNA 1207-5P and its host gene, the long non-coding RNA Pvt1, as mediators of extracellular matrix accumulation in the kidney: implications for diabetic nephropathy. PLoS ONE.

[146] Li J, Zhao Q, Jin X, Li Y, Song J (2020). Silencing of LncRNA PVT1 inhibits the proliferation, migration and fibrosis of high glucose-induced mouse mesangial cells via targeting microRNA-93-5p. Biosci Rep.

[147] Matouk IJ, DeGroot N, Mezan S, Ayesh S, Abu-lail R, Hochberg A, Galun E (2007). The H19 non-coding RNA is essential for human tumor growth. PLoS ONE.

[148] Tremblay KD, Saam JR, Ingram RS, Tilghman SM, Bartolomei MS (1995). A paternal–specific methylation imprint marks the alleles of the mouse H19 gene. Nat Genet.

[149] Okamoto K, Morison IM, Taniguchi T, Reeve AE (1997). Epigenetic changes at the insulin-like growth factor II/H19 locus in developing kidney is an early event in Wilms tumorigenesis. Proc Natl Acad Sci U S A.

[150] Xie H, Xue JD, Chao F, Jin YF, Fu Q (2016). Long non-coding RNA-H19 antagonism protects against renal fibrosis. Oncotarget.

[151] Shi S, Song L, Yu H, Feng S, He J, Liu Y, He Y (2020). Knockdown of LncRNA-H19 ameliorates kidney fibrosis in diabetic mice by suppressing miR-29a-mediated EndMT. Front Pharmacol.

[152] Yi H, Peng R, Zhang LY, Sun Y, Peng HM, Liu HD, Yu LJ (2017). LincRNA-Gm4419 knockdown ameliorates NF-κB/NLRP3 inflammasome-mediated inflammation in diabetic nephropathy. Cell Death Dis.

[153] Ji TT, Wang YK, Zhu YC, Gao CP, Li XY, Li J, Bai F (2019). Long noncoding RNA Gm6135 functions as a competitive endogenous RNA to regulate toll‐like receptor 4 expression by sponging miR‐203‐3p in diabetic nephropathy. J Cell Physiol.

[154] Li SY, Susztak K (2016). The long noncoding RNA Tug1 connects metabolic changes with kidney disease in podocytes. J Clin Invest.

[155] Long J, Badal SS, Ye Z, Wang Y, Ayanga BA, Galvan DL, Green NH (2016). Long noncoding RNA Tug1 regulates mitochondrial bioenergetics in diabetic nephropathy. J Clin Investigation.

[156] Duan LJ, Ding M, Hou LJ, Cui YT, Li CJ, Yu DM (2017). Long noncoding RNA TUG1 alleviates extracellular matrix accumulation via mediating microRNA-377 targeting of PPARγ in diabetic nephropathy. Biochem Biophys Res Commun.

[157] Yan B, Yao J, Liu JY, Li XM, Wang XQ, Li YJ, Tao ZF (2015). lncRNA-MIAT regulates microvascular dysfunction by functioning as a competing endogenous RNA. Circ Res.

[158] Zhou L, Xu D, Sha W, Shen L, Lu G, Yin X (2015). Long non-coding MIAT mediates high glucose-induced renal tubular epithelial injury. Biochem Biophys Res Commun.

